# Zebrafish: A Versatile Animal Model for Fertility Research

**DOI:** 10.1155/2016/9732780

**Published:** 2016-07-31

**Authors:** Jing Ying Hoo, Yatinesh Kumari, Mohd Farooq Shaikh, Seow Mun Hue, Bey Hing Goh

**Affiliations:** ^1^Biomedical Research Laboratory, Jeffrey Cheah School of Medicine and Health Sciences, Monash University Malaysia, Jalan Lagoon Selatan, 47500 Bandar Sunway, Selangor Darul Ehsan, Malaysia; ^2^School of Science, Monash University Malaysia, Jalan Lagoon Selatan, 47500 Bandar Sunway, Selangor Darul Ehsan, Malaysia; ^3^Sunway College, Jalan Universiti, Bandar Sunway, 46150 Petaling Jaya, Selangor Darul Ehsan, Malaysia; ^4^Neuropharmacology Research Laboratory, Jeffrey Cheah School of Medicine and Health Sciences, Monash University Malaysia, Jalan Lagoon Selatan, 47500 Bandar Sunway, Selangor Darul Ehsan, Malaysia; ^5^Novel Bacteria and Drug Discovery Research Group, School of Pharmacy, Monash University Malaysia, Jalan Lagoon Selatan, 47500 Bandar Sunway, Selangor Darul Ehsan, Malaysia; ^6^Center of Health Outcomes Research and Therapeutic Safety (Cohorts), School of Pharmaceutical Sciences, University of Phayao, Phayao 56000, Thailand

## Abstract

The utilization of zebrafish in biomedical research is very common in the research world nowadays. Today, it has emerged as a favored vertebrate organism for the research in science of reproduction. There is a significant growth in amount numbers of scientific literature pertaining to research discoveries in reproductive sciences in zebrafish. It has implied the importance of zebrafish in this particular field of research. In essence, the current available literature has covered from the very specific brain region or neurons of zebrafish, which are responsible for reproductive regulation, until the gonadal level of the animal. The discoveries and findings have proven that this small animal is sharing a very close/similar reproductive system with mammals. More interestingly, the behavioral characteristics and along with the establishment of animal courtship behavior categorization in zebrafish have laid an even stronger foundation and firmer reason on the suitability of zebrafish utilization in research of reproductive sciences. In view of the immense importance of this small animal for the development of reproductive sciences, this review aimed at compiling and describing the proximate close similarity of reproductive regulation on zebrafish and human along with factors contributing to the infertility, showing its versatility and its potential usage for fertility research.

## 1. Introduction

*Danio rerio*, or commonly known as zebrafish, is a tropical freshwater fish. It was previously a well-known aquarium fish at home, which has rapidly transformed into an indispensable animal model for scientists of today's world. The numerous advantages and characteristics possessed by this small animal have never failed in tempting researchers in utilizing this animal model for their scientific research projects. Perhaps, the popular usage of this animal owns to their cheap and easy maintenance of animal in the laboratory [1, 2]. Nonetheless, the fact that well-characterized gene functions of zebrafish which is showing a high degree of similarity with human gene have certainly improved confidence level and potential implications of research findings [3, 4]. This may explain the drastic usage growth of this small animal in experimentation in recent years. Basically, the studies which have been done with zebrafish had basically contributed to a vast advancement in many fields of science. The usage of zebrafish in scientific research could be seen playing significant roles in fundamental research such as evolutionary science, genetics, neurobiology, and development biology [5–7]. In terms of application sciences, it has been widely utilized for drug discovery or even environmental monitoring effort [8–10].

Fertility or reproductive science is one of the popular fields in medical research. The impactful discoveries in this field are including the assisted reproductive technology (*in vitro* fertilization), erectile dysfunction medication such as cGMP-specific phosphodiesterase, and hormonal treatment techniques to overcome infertility [11, 12]. Unfortunately, infertility problems are still persisting around the globe with an alarming percentage of around 20% of infertile couples [13]. This thus explained that the need of continuous research in fertility and further advancement in level of fundamental understanding of the reproductive system in human is a must in general.

In this context, zebrafish have swum into view as a promising model in assessing reproductive complications owing to its developmental and physiological advantages [14–17]. The short cycle of reproductive period and the transparency of these animals at early developmental stages are allowing the researchers to carry out research activities in more efficient or hassle-free way than before [18, 19]. A close degree of similarity of reproductive regulation systems between human and zebrafish has also permitted the researchers to study and understand the system in a more comprehensive way. This could be also seen from the identification of important neurons which are involved in regulating the reproductive system and presence of similar reproductive hormones and responses in this animal [20, 21]. Along with these fundamental research findings, zebrafish have indirectly granted the scientists evaluating the potential hazardous compounds on reproductive system on human. Furthermore, zebrafish are amenable to genetic manipulation which has offered another important aspect for researchers to study the gene effects on reproduction [22, 23]. Together with the establishment of courtship behavior in zebrafish [1, 24], it dispelled the pervasive myths of zebrafish usage in fertility research.

## 2. Reproductive Gender and Biology of Zebrafish

Mammals have dimorphic sex chromosomes and practice male heterogametic system. Gene* SRY (sex determining region Y)* is of large effect on mammals' sex determination by acting as a genetic switch that initiates male pathway in bipotential gonad [25, 26]. Zebrafish, however, lack of the sex determination cascade. Complex sex determination system with combined effects of genetic and environmental factors such as surrounding temperature [27], exposure to sex hormones (e.g., oestrogen and androgen), and oxygen availability [28] have been revealed by consistent works in gonad ontogenetic differentiation of zebrafish. On the genetic point of view, recent studies have suggested chromosome 4 as the potential sex chromosome in natural zebrafish with their sex determination mechanism strongly weakened in domesticated zebrafish strain [29, 30].

On the other hand, similar to humans, several autosomal genes have proven significant roles in development and differentiation of gonads and reproductive cells. For instance, Anti-Müllerian Hormone (*amh*) is one of the critical hormones in sex differentiation during fetal development. Under tight transcriptional regulation by* sox9*, steroidogenic factor 1 (*SF-1*), Wilm's tumor suppressor gene 1 (*wt1*), and* GATA4*,* amh* is released from the Sertoli cells in fetal testes [31–33]. In addition to degeneration of Müllerian ducts, a pair of ducts which further develops into Fallopian tubes and uterus,* amh* also inhibits the expression of a P450 aromatase enzyme, known as* Cyp19a1*, which converts androgens to estrogens [34]. In this context, zebrafish share similar features of vertebrate gonadogenesis by having* amh* expression in their gonad along with the identification of gene binding sites for the same transcriptional factors in the* amh* gene promoter sequence [35, 36]. Besides, inhibition of early spermatogonial differentiation remains as the other known aspect of conserved bioactivity of* amh* between zebrafish and mammals [37].

Meanwhile, zebrafish have short generation time by having all of the precursors for major organs after 24 hours of fertilization and typically achieve reproductive maturity within 3 to 6 months after fertilization with the maturity period corresponding to the body length of approximately 23 mm [24]. Although mice have similar development length, zebrafish which are oviparous can produce around 200 to 300 eggs per week, thus permitting large-scale experimental analysis. High level of genetic homology is also shared across both species [3]. On the other hand, zebrafish display similar anatomy of germ cell organs to that in humans [38, 39]. Male zebrafish have paired testes with tubule organizations. Within each tubule, the walls are lined by Sertoli cells and they function mainly to support testes morphogenesis and spermatogenesis while Leydig cells detected in the interstitial spaces act as primary testosterone producer [38, 39]. One distinct spermatogenesis pattern observed in zebrafish is the presence of spermatogenic cyst which consists of a group of Sertoli cells enveloping germ cells that develop synchronously, instead of having few germ cells with different development stages in Sertoli cell as observed in higher vertebrates [40]. On the other hand, study also showed the presence of accessory sperm duct gland in male zebrafish which functions mainly in the secretion of mucosubstances and production of sperm trails [41].

While for female zebrafish, the key similarities of the reproductive system lie in the structure and functions of ovaries. A pair of bilateral ovaries is observed in female and it is located between the swim bladder and abdominal wall [42]. Ovarian wall is lined with thin epithelium with numbers of oogonia and oocyte follicles surrounded by interstitial tissues and somatic cells observed. Lobulated structures with interlobular spaces and the joining with oviduct have been revealed through histological sectioning [43]. Across vertebrates, ovaries are the site of development and production of female gametes [44, 45]. There are four stages of ovarian development in zebrafish, namely, primary oocyte stage with observation of relatively small spherical cells, cortical-alveolar stage with enlarged oocytes filled with cortical alveoli, vitellogenic stage characterized by presence of egg yolk in oocytes, and finally maturation stage in which oocytes with irregular layer can be observed [46]. Similar to other teleost fish and humans, zebrafish follicle contains an oocyte surrounded by zone radiata along with a follicular layer made up of inner granulosa cells and outer thecal cells layer [47]. Ovulation takes place following rupture of the layers and it is mainly induced by male gonadal pheromones [24, 44]. It is also significantly promoted with the accumulation of steroid glucuronides such as 5a-androstane-3a, l7*β*-diol, and cholesterol in male holding water and administration of testes homogenates [38, 48].

Altogether, besides the biological advantages of zebrafish which include rapid embryonic development, large embryonic production, and high degree of similarity to human genome, there are striking homologies between the reproductive system of human and zebrafish and the many similarities in aspects spanning from the reproductive anatomy and physiology to gene functions and expression. As such, they serve as the ideal system for analyzing fertility as well as embryonic development.

### 2.1. Reproductive Behavior and Performance of Zebrafish

Zebrafish are early morning breeders and group spawners [24, 49]. Females proved capable of spawning at frequent but irregular basis, with several hundred of eggs in a spawning session [50]. An interspawning frequency of approximately one to six days is observed [51]. Eggs spawned by zebrafish are optically translucent and are normally larger as compared to other fishes, with approximately 0.7 mm in diameter [24]. Besides having healthy sexual organ and morphological sexual characteristics development and undisrupted steroidogenesis [52, 53], normal courtship behavior is one of the crucial criteria for successful reproduction among zebrafish [24]. Both male and female zebrafish display different mating behavior. The five typical behavior displayed by male zebrafish are chase in the form of swimming or following the females (chase), having contact with female by using its nose or tail (tail-nose), circling around females (encircle), circling around females in the “figure eight” pattern (zig-zag), and rapid tail movement against females' bodies (quiver) [1, 24, 54]. While for females, their sexual behaviors begin with approach by swimming abruptly towards males (approach), swimming alongside males or staying still when being chased (escort), swimming around males or halting in front of males (present), and swimming to one preferred location in its habitat (lead) and oviposition (egg-lay) [1, 24].

During a courtship episode, chase, tail-nose, and approach are the three initiatory mating activities displayed by both genders of zebrafish followed by present and escort from females as receptive behavior [1, 24]. However, some females may chase males away aggressively when the male's approach is unfavorable. Then, repetitive behaviors such as encircle and zig-zag are presented [1, 24]. After the display of repetitive behavior, female zebrafish start to swim towards a specific location for at least three times [49]. Finally, males swim and spread their caudal and dorsal fins around females for alignment of their genital pores. Rapid tail oscillation can then be observed to encourage spawning [1, 49, 54]. Studies suggested the simultaneous release of sperms and eggs. To be precise, sperms are released before egg deposition [41]. Generally, male courtship behavior peaks in the first 30 minutes of courtship period and it may continue for an hour [1]. For both territorial and nonterritorial males, the same courtship behavior can be identified. However, nonterritorial males tend to pursue females in the whole available mating space whereas territorial males display their mating behavior limited to the areas close to spawning site and other males' approaches are often unwelcomed [55].

Reproductive performance of zebrafish is affected by several environmental factors such as photoperiod [56, 57], tank volume [58], water temperature and pH [57], topography, fish densities, and presence of natural habitats items such as aquatic plants and substrates [59]. Zebrafish have endogenous reproduction rhythm which is significantly influenced by photoperiod and a cycle of 10-hour light and 14-hour dark has been normally practiced for breeding [56, 57]. In both wild and laboratory environments, zebrafish normally spawn in the first few hours of daylight [1, 24]. However, spawning in the afternoon by wild zebrafish and in the late evening by zebrafish in captivity have also been observed [24]. Additionally, they prefer to spawn in the areas with natural habitats items such as aquatic plants and substrates as well as in shallow areas with greater embryo production observed [59]. On the other hand, chamber volume varies according to the number and size of breeding adults. A tank volume of not less than 300 mL is recommended for successful breeding between six zebrafish with weight ranges from 0.50 g to 0.70 g and 0.95 g for male and female, respectively [58]. Meanwhile, zebrafish breeders normally go with a water temperature of 24 to 30°C along with pH between 7.0 and 8.0 [57]. Feeding practices which include type of diet, frequency, and density of feeding are also of significant importance in zebrafish spawning. Several recommended diets for breeding zebrafish have been suggested. These include feeding zebrafish with formulated diet, Gemma Micro 300 at 5% of body weight once daily [60], flake diet to satiation three times daily or on a rotating diet of flake food and freshly hatched brine shrimp (*Artemia nauplii*) in every morning and evening, respectively [61, 62], and* Spirulina platensis*-based diet three times daily at 5% of body weight [63]. Meanwhile, nutritional supplementation in phospholipids (phosphatidylcholine) [64], highly unsaturated fatty acids (e.g., diet with 1 : 1 squid oil : linseed oil) [65] and* Moringa* leaf [66] have been proved to promote reproductive system of zebrafish. It is important to note that breeding zebrafish require rich feeding.

Phenotypic cues such as paternal and maternal body size [51, 67, 68], fin length [69, 70], group size [71], and behavioral traits [72] have been extensively studied to identify their potential effects on the reproductive success of zebrafish. Besides the well-known fact that large females displayed higher fecundity along with provision of high qualities of eggs and larvae [51, 67], pronounced size-dependent paternal effect on a broad range of reproductive parameters is identified. Large (28-29 mm) and very large (30-31 mm) males can contribute to higher hatching probability along with early hatching time and larger offspring hatched [51, 68]. In an indirect male-size effect, females have shown their preference towards large (26–34 mm), territorial males by allocating more eggs to them as compared to small males [24, 51, 73]. Meanwhile, studies showed that wild type females do not show preferences towards long and short fin males, hence suggesting that it is the total body size that females prefer as compared to overall apparent size [69, 70]. However, one study discovered the strong association between long fin males and females [70]. Besides visual information, adult females display mate selection in response to olfactory cue. They showed strong preference towards odour stimuli from nonkin males, thus avoiding inbreeding which often leads to reduced fecundity and quality of offspring [74]. On the other hand, lower reproductive success in terms of mean per capita egg production was observed at higher fish densities (e.g., 5 males and 10 females), owing to increased aggression level among males and competition among females over oviposition site [71]. On top of that, decreased courtship rate was shown in the high density male-biased group. This observation can be explained by the tendency of territorial males to engage in territorial defense, rather than in mate acquisition [24, 71]. In view of the significant impact of population density and sex ratio on mating success of zebrafish, a small mating group of approximately five along with male to female ratio of 1 : 2 is often recommended for effective breeding [71]. During a courtship period, males often compete with each other. Besides acquiring a territory and maintaining dominancy, study illustrated that males that are bold and aggressive have greater reproductive fitness by allowing greater proportion of eggs fertilized [72].

## 3. Regulation of Reproductive System

The reproductive system is a functional cooperation among sex organs in an organism to produce a new life. In general, gametes producing gonads, ducts, and openings are some of the main reproductive elements shared among vertebrates [75]. Normal sexual functioning requires strong genital muscles, extensive vascular network, and tight neuroendocrine regulations. In mammals, the reproductive system is tightly regulated by three interrelated hormonal feedback control axes: hypothalamic-pituitary-adrenal (HPA) axis, hypothalamic-pituitary-gonadal (HPG) axis, and hypothalamic-pituitary-thyroid (HPT) axis [75]. The key components and functions of all of the three axes in zebrafish correspond closely to mammals [21].

### 3.1. Hypothalamic-Pituitary-Gonadal (HPG) Axis

Hypothalamic-Pituitary-Gonadal (HPG) axis is defined as a functional cooperation between three endocrine glands: hypothalamus, anterior pituitary gland (APG), and gonads in regulating reproduction, development, and aging in animals [76]. In HPG axis, kisspeptin (Kiss1) neurons, and GnRH neurons are the two main control points in hypothalamus [77]. Both of the neurons play important role in the regulation of the secretion of reproductive hormones, luteinizing hormone (LH), and follicular stimulating hormone (FSH) from APG [78, 79].

Evolutionary studies showed the presence of four Kiss-R genes lineages (Kiss-R1a, Kiss-R1b, Kiss-R2a, and Kiss-R2b). In humans, only Kiss-R1a lineage is conserved [77]. Kiss1 neurons are the main mediator of sex steroid feedback loop [80, 81]. Along with this, sex steroid receptors such as estrogen receptor alpha receptors, androgen receptors, and progesterone receptors can be found on the neurons. As the neurons are colocalized with GnRH neurons, they are also defined as the upstream regulator of GnRH neurons in hypothalamus [82]. In humans, there are more Kiss1 neurons in the arcuate (ARC) nucleus as compared to the anteroventral periventricular nucleus (AVPV) [83]. Kiss1 neurons located in ARC act as the regulator in negative feedback mechanism of sex steroid hormones on the secretion of GnRH from GnRH neurons. While for Kiss1 neurons in AVPV, they mainly function in the preovulatory GnRH/LH surge process [82, 84].

To date, zebrafish remains as one of the few teleosts with detailed information gathered on the distribution and functions of kisspeptin. In zebrafish, two kiss genes, Kiss1 and Kiss2, have been identified successfully through* in situ* hybridization [85, 86]. Kiss1 neurons are limited to habenular nucleus while Kiss2 are widely distributed in ventral and caudal region of hypothalamus, thalamus, preoptic area, mesencephalon, and pallium [87]. Meanwhile, few studies have reported the functional similarities of neuropeptide kisspeptin between zebrafish and mammals. Similar to mammalian Kiss1 signaling, habenular Kiss1 in zebrafish plays pivotal role in puberty onset through regulation of GnRH secretion [88, 89]. Additionally, regulation of gonadotropins release is also one of the potential physiological roles of kisspeptin in both zebrafish and human [83, 85]. Nevertheless, Kiss2 but not Kiss1 appears as the predominant GTH-I and GTH-II regulator in zebrafish [88].

GnRH neurons are neuron cells that play pivotal role in regulation of the release of reproductive hormones, LH and FSH from APG [79, 90]. Three types of GnRH genes: herring GnRH (GnRH1), chicken GnRH-II (cGnRH-II), and salmon GnRH (sGnRH/GnRH3) are identified in humans [91]. GnRH1 is the classical hypothalamic reproductive neuroendocrine factor which further allows LH and FSH secretion from APG. In zebrafish, two forms of GnRH are identified, namely, chicken GnRH-II (cGnRH-II) in the midbrain tegmentum and salmon GnRH (sGnRH/GnRH3) which is expressed in olfactory bulb and preoptic area of hypothalamus [92, 93]. Unlike other fishes which possess three GnRH isoform [94], GnRH3, instead of GnRH1 takes the role of activating and controlling the pituitary release of LH and FSH in zebrafish [95].

In both humans and zebrafish, FSH (GTH-I in zebrafish) and LH (GTH-II in zebrafish) are produced by pituitary cells in response to GnRH from GnRH neurons in hypothalamus [83, 95]. The roles of these glycoprotein hormones in steroidogenesis and gametogenesis are conserved across species. In males, FSH regulates the spermatogonial proliferation and differentiation in Sertoli cells. While for females, FSH plays critical role in stimulating estrogen and inhibin alpha subunit (inha) production during folliculogenesis, as also reported in mammals [96, 97]. In mammals and zebrafish, expression of inha peaks during full-grown stage of follicles and it acts as an endocrine hormone which triggers final oocyte maturation and ovulation by stimulating LH production [98, 99]. In regard to the characterization of FSH receptors, zebrafish GTH-I receptors display strong sequence similarities to that of humans [100]. Besides FSH, oocyte maturation and growth in both humans and zebrafish require the hormonal functions of LH and 17*α*,20*β*-dihydroxy-4-pregnen-3-one (17*α*,20*β*-DP), a maturation inducing hormone [101, 102]. Meanwhile, LH also regulates steroidogenesis in Leydig cells, though to a lesser extent in zebrafish [103]. Nevertheless, cAMP/protein kinase A pathway remains as the common underlying mechanism in LH-mediated testicular steroid production across species [103]. Viewing from the findings above, HPG axis of zebrafish appears to have striking resemblance to that of more evolved vertebrates, conserving the major outline of reproductive cells and hormones identified in mammals.

### 3.2. Hypothalamic-Pituitary-Thyroid (HPT) Axis

**Figure 1:**
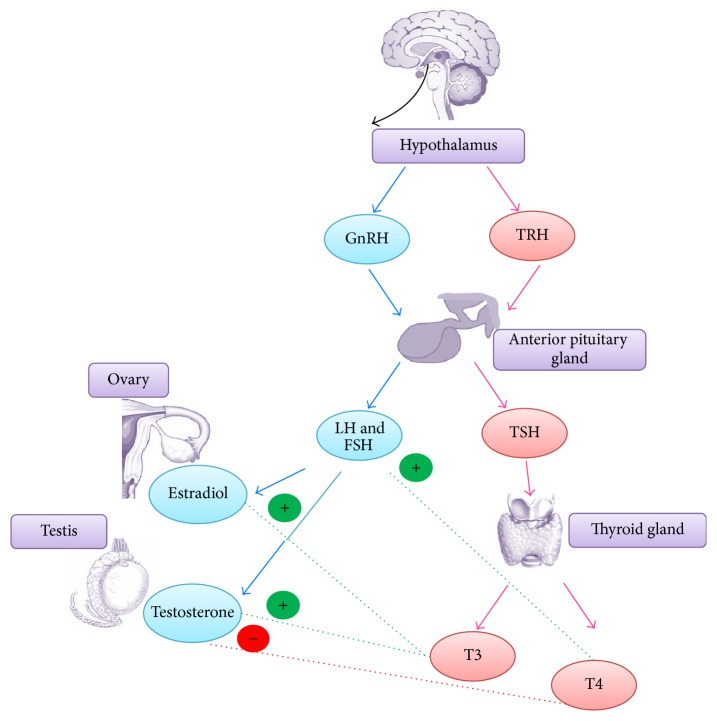
Crosstalk between HPG and HPT in mammals. T3 displays its estrogenic action by stimulating the expression of both estrogen receptors *α* and *β*. Increased in expression of steroidogenic acute regulatory protein (StAR) followed by elevated testosterone level were also observed following acute exposure to T3. Meanwhile, administration of T4 has been shown to cause elevation in the level of LH and FSH. However, low serum testosterone was observed under T4-induced hyperthyroidism and the low testosterone level is attributed to the decreased catalytic activities of testicular enzymes involved in lipogenesis (blue arrow: HPG; pink arrow: HPT).

HPT is physiologically related to HPG and both of the axes work together in regulating reproductive functions [104]. The presence of thyroid hormone receptors in ovaries and effect of estrogen hormone level on HPT axis have proven the reciprocal relationship between these two axes [75, 105]. In mammals, triiodothyronine (T3) and tetraiodothyronine (T4) are the two principal thyroid hormones secreted from the thyroid gland, a butterfly-shaped organ located in the neck. The hormonal output of thyroid gland is regulated by thyroid stimulating hormone (TSH) secreted from APG, which itself is controlled by thyroid releasing hormone (TRH) from hypothalamus [106]. The main function of thyroid system is to regulate the metabolism, growth, and development of an individual. On the other hand, the amounts of thyroid hormones secreted are known to affect the release of reproductive hormones such as LH, FSH, and several steroid hormones, thus being consistent with the crosstalk concept between HPT and HPG [107–110] (Figure 1).

As the reproductive system is tightly regulated by HPT, several reproduction disorder symptoms will be displayed when the axis is disrupted [111–113]. With the excessive secretion of thyroid hormones from thyroid gland, or otherwise known as hyperthyroidism, women often experience menstrual disturbance and anovulatory cycles. In terms of menstrual disorder, amenorrhea and oligomenorrhea have been often reported, as well as a changes in the amount of menstrual flow such as hypomenorrhea and hypermenorrhea [114, 115]. On the other hand, semen quality is adversely affected in hyperthyroidism men [112, 116]. While for hypothyroidism, delayed puberty can be detected among teenagers and mature women tend to suffer from abnormal menstrual cycles and increased risk of fetal wastage [111, 113]. Following the decrease in the amount of LH, FSH, sex hormone binding protein, and serum testosterone level in hypothyroidism men, increased testicular size and decreased sperms qualities in terms of sperm morphology and motility and semen volume have been elucidated in several studies [117, 118].

**Figure 2:**
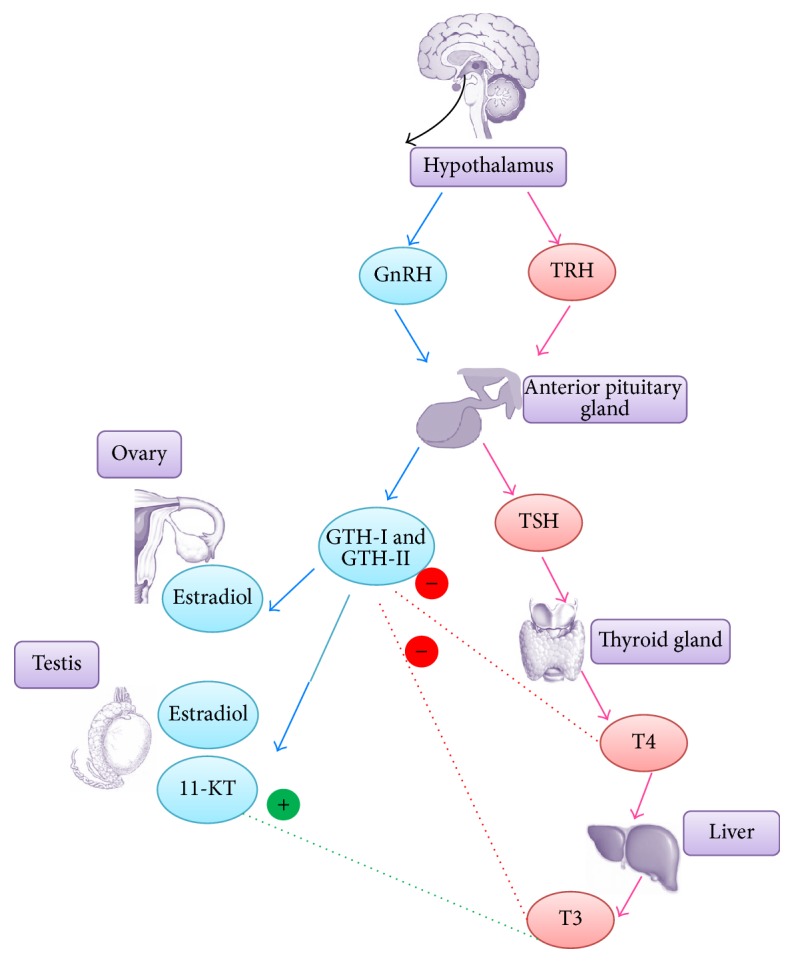
Crosstalk between HPG and HPT in zebrafish. Together with GTH-I, T3 elevates the expression of ar and cyp17a1, leading to increased 11-KT production and sensitivity in zebrafish testicular tissues. Meanwhile, T3 alone plays stimulatory role in the proliferation of Sertoli cells and type A undifferentiated spermatogonia. However, concentrations of thyroid hormones were negatively correlated with amount of GTH-I and GTH-II (blue arrow: HPG; pink arrow: HPT).

In many aspects, thyroid system in teleost, particularly zebrafish, is similar to the mammalian system. Zebrafish apparently have thyroidal tissues with the same origin as those of mammals. Genes responsible for thyroid development such as* pax2a* and* pax8* and* nkx2.1a* and* hhex* are conserved between zebrafish and mammals [119, 120]. Meanwhile, release of TSH from APG followed by synthesis of T3 from thyroid glands is observed in both organisms [121]. The fundamental roles of thyroid hormones in regulating metabolism, early development, and differentiation of zebrafish correspond closely to thyroidal hormones functions in mammals. Additionally, alteration in the amount of thyroidal hormones secreted in zebrafish affects the regulation of reproduction system too (Figure 2). In male zebrafish, T3 stimulates mitotic activities in Sertoli cells as well as proliferation of type A undifferentiated spermatocytes [40]. The recent studies have also shown that the change in concentrations of T3 and T4 may affect the levels of GTH-I and GTH-II, which are known to play important roles in stimulation of steroidogenesis and gametogenesis [52, 75]. Additionally, hyperthyroidism in larval zebrafish is shown to result in decreased aromatase activity along with estrogen synthesis, leading to testicular formation and skewed sex ratio in favor of males [122]. The observed reproductive physiological changes under hyperthyroid condition are consistent across a range of animal models which includes mammals and reptiles, thus lending support to the statement regarding the masculinizing effect of thyroid hormones [123–125]. The combined potentiation effect of T3 and GTH-I on the androgen biosynthesis and sensitivity of testes further suggested the crosstalk between HPT and HPG in zebrafish [126]. Altogether, the findings obtained on the components and reproductive functions of HPT axis reveal many parallel between zebrafish and human and this further delineates the remarkable potential of zebrafish as the animal model in infertility studies.

### 3.3. Hypothalamic-Pituitary-Adrenal (HPA) Axis

HPA axis is the complex set of interaction between three—hypothalamus, pituitary gland, and adrenal gland [127]. It is the major constituent of neuroendocrine system which produces stress and mood responses and involves in the regulation of immune and reproductive system [128]. Under stress condition, neuroendocrine neurons in the paraventricular nucleus of hypothalamus are stimulated to produce corticotrophin-releasing hormone (CRH) and vasopressin [129]. These two hormone peptides in turn lead to the secretion of adrenocorticotropic (ACTH) hormones from APG. Biosynthesis of several corticosteroids such as cortisol can be observed following blood transportation of ACTH from APG to adrenal cortex [130]. Cortisol is the steroid hormone produced from the zone fasciculate of the adrenal cortex in response to stress and low sugar level condition. Under stressful condition, the physiological demands for energy can be met through increased gluconeogenesis process stimulated by cortisol [130]. At the meantime, cortisol prevents overactivation of immune system and inflammation during stress by allowing the shift towards type 2 helper T cells (Th2) immune response [130].

In zebrafish, stress axis is known as hypothalamus-pituitary-interrenal (HPI) axis [131]. The anatomy and physiology of the pituitary are highly conserved between zebrafish and mammals. Similar to mammals, pituitary in zebrafish appears in two different parts with distinct functions. The hormones produced by pituitary glands under stress are the same as mammals [132, 133]. The pituitary-secreted stress hormones, ACTH, will then bind to type 2 melanocortin receptor (MC2R) located in the interrenal gland of zebrafish, the homolog of mammalian adrenal gland [134]. Meanwhile, evolutionary conservation of MC2R trafficking and signaling was observed in zebrafish, particularly in terms of the presence of three forms of melanocortin 2 receptor accessory proteins (MRAP) and their structural features and the critical roles of MRAP 1 in MC2R signaling following ACTH stimulation and MRAP 1 or MRAP 2a in localization of MC2R to plasma membrane [135–137]. Across both species, cortisol is the main corticosteroid produced under stress condition. Cortisol stress signaling is primarily mediated by glucocorticoid receptor (GR), a ligand-activated transcription factor. In this context, studies suggested the presence of single GR gene with two splicing variants, termed GR*α* and GR*β* in zebrafish, which shows high similarity level to its human equivalent [134, 138–140].

## 4. Infertility

Infertility is defined as the incapability of an individual to achieve clinical pregnancy despite having regular unprotected sexual intercourse for more than 12 months. Epidemiology study showed that approximately 20% couples worldwide are suffering from infertility [13]. In general, infertility is caused by male factors such as poor sperm qualities and quantities [141], female factors such as abnormal ovulation and tubal pathology [142, 143], combined male and female factors, and unexplained infertility factors [144]. Hormonal imbalance, particularly due to unhealthy and stressful lifestyles [145, 146], and prolonged exposure to harmful chemicals and unfavorable environmental conditions [147–149] are some of the suggested underlying pathogenic mechanisms in infertility.

### 4.1. Stress-Induced Infertility

When zebrafish are exposed to stressor, nucleus preopticus (NPO), a region homologous to paraventricular nucleus (PVN) in hypothalamus of mammals, will secrete CRH. In response to CRH, corticotrophs in APG will release ACTH, the hormones which further stimulate cortisol biosynthesis in interrenal gland [132, 133]. The influence of HPI on reproductive axis in zebrafish is similar to that of mammals. The secretions of biological hormones such as CRF, ACTH, and cortisol under stress generally lead to impaired reproductive system through inhibition of the release of reproductive hormones and gametogenesis (Table 1) [150]. In female zebrafish, the disruptive effects of ACTH and cortisol on gametogenesis and fertilization success have been illustrated through the identification of oocytes with DNA damage as well as reduced nucleic acid via disruption of protein synthesis [151]. Additionally, ACTH induces strong vacuolization in zebrafish ooplasm and similar condition was also observed in mammalian adrenal gland cells following exposure to ACTH [151, 152]. On top of that, ACTH suppresses gonadotropin-stimulated estradiol release from ovarian follicles [150]. This stress-induced inhibition of steroidogenesis may be related to the binding of ACTH to melanocortin 2 receptor (MC2R), a specific ACTH receptor identified in zebrafish ovary along with the presence of inhibitory G protein in MC2R signaling [150]. To the best of our knowledge, currently there is no study identified on the effect of ACTH on male reproductive system. Nevertheless, MC2R receptors have been identified in male gonads and hence leading to the hypothesis that ACTH may involve in male gonadal steroid modulation too.

**Figure 3:**
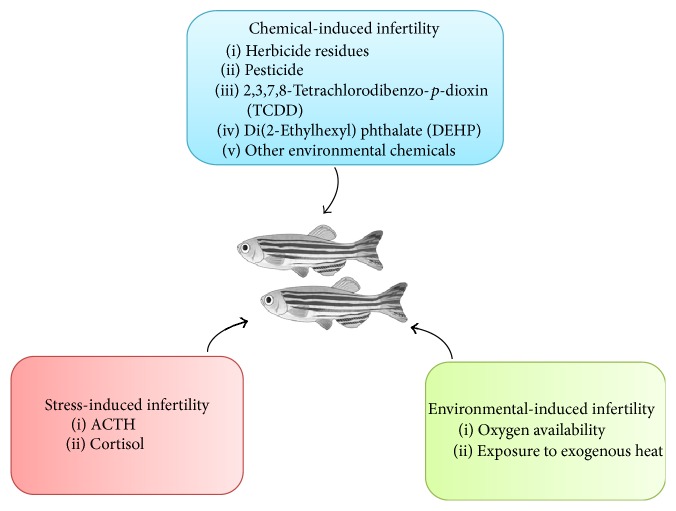
Infertility factors in zebrafish.

Following the high similarities identified in zebrafish HPG and HPA regulatory axis as compared to human, the reproductive health status of zebrafish under stress is highly predictive of mammalian responses and hence further strengthen the potential of zebrafish as research model in infertility studies (Figure 3).

### 4.2. Chemical-Induced Infertility

Since the beginning of industrial era in around 1750, a sharp increase in the amount of chemicals produced and released in the surrounding environment has been observed [153]. At the same time, there is significant increase in the health threat following chemical exposure, leading to the increasing demand for robust and cost effective methods to assess the chemical effects in human health, particularly growth and development along with reproductive system [154, 155]. As aforementioned, mammals such as rat and mice have been normally used to assess the reproductive toxicity of chemicals. Unfortunately, mammals-based assays to assess reproductive toxicity are time-consuming, complex, and expensive to have large-scale experimental analysis [156]. Moreover, high dosages are often required for mammal experimentation, thus leading to unpredictable toxicity levels of the environmental chemicals as the concentration levels of chemicals in the environment are often low [157]. Hence, zebrafish are recommended as the model system in this research field, particularly for water-soluble pollutants following the ease of chemical introduction into zebrafish [158, 159] and increased throughput within a shorter research period (Figure 3).

#### 4.2.1. Herbicide Residues (Glyphosate)

Focusing on glyphosate, or commercially known as Roundup, it is a chemical formulation in herbicide that has been used extensively in agricultural field worldwide and emerged in the topping list of herbicide usage in Western countries since 1974 [160]. It controls the plants population by acting as an inhibitor for enzyme 5-enolpyruvylshikimate-3-phosphate synthase, an enzyme which catalyzes the production of intermediate in the plant biosynthesis of aromatic amino acids process [161]. Although this biosynthesis pathway is absent in animals, studies have shown the reproductive adverse effects of glyphosate in a range of organisms, particularly aquatic organisms [162–164]. This water-soluble pollutant eventually affects human health, especially the sexual and reproductive development via consumption of contaminated food and drink [165–167].

Viewing the high structural similarities in the reproductive axis of zebrafish as compared to humans, zebrafish are often utilized as the model in the assessment of reproductive toxicity of environmental chemicals, including herbicides. Following exposure to high concentration of glyphosate, significant increase in expression of* cyp19a1* gene, aromatase activity, and the predominant estrogen receptor in ovary,* esr1*, was identified, thus revealing the potential steroidogenesis disruption effect of glyphosate in zebrafish [164]. It is hypothesized that the increased* cyp19a1* and* esr1* expression are compensatory mechanisms in ovary to restore the balance of estrogen hormone level [164]. Similarly, a number of* in vitro* studies have revealed the potential of glyphosate as endocrine disruptor via inhibition of aromatase activities in human cell lines [168, 169]. The disruption of steroidogenic biosynthesis pathway was hypothesized as one of the major underlying factors which contributed to reduced egg productions along with histological evidence of ovarian follicle atresia in adult female zebrafish [164]. Meanwhile, steroid hormone biosynthesis in testes was also affected. Upregulation of antioxidant genes and presence of sperms with lowered membrane and DNA integrity and motility were also observed in glyphosate-exposed adult male zebrafish, suggesting the potential of glyphosate in inducing oxidative stress in the testis [161]. High parental exposure to glyphosate eventually caused increase in mortality rate of embryo during early development and this finding is generally in accordance with evidence from other species such as mammals [170] and amphibians [171].

#### 4.2.2. Pesticides (Endosulfan)

Besides herbicide, aquatic environments are facing persistent pesticide pollution. A mixture of endosulfan I and II is often included in the pesticide formulation [172]. Once it is released into the aquatic environment through field runoff and atmosphere transport, it exists in the form of endosulfan sulfate and diol in aquatic sediments and water, respectively [173, 174]. All these compounds are further broken down into alcohol, hydroxyl, ether, hydroxyl ether, and lactone [175]. Endosulfan sulfate is the only toxic breakdown product and has longer half life up to years. Endosulfan is proven to be bioaccumulative and has potential effect on the reproductive performance, primarily via disruption of endocrine functions [175].

Ova-testes status, testicular damage, and sperms necrosis were observed among exposed adult male zebrafish at a very low concentration of endosulfan (10 ng/L) [176]. The pathological changes in testes were highly correlated with the decreased hatching rate [176, 177]. Additionally, studies have proposed the binding ability of endosulfan to estradiol receptors found on liver, thus leading to increased vitellogenin level in male zebrafish [176]. At the mean time, degenerative changes such as increased sizes of follicular cells, oocyte membrane folding, and reduced vitellogenesis can be observed on atretic follicles in female zebrafish [176]. Besides, delayed sexual maturity and reduced spawning frequency were also observed [178]. Altogether, reproductive toxicities of endosulfan, which include DNA damage and induction of oxidative stress [179, 180], developmental abnormalities [181], and histopathological changes of organs [182, 183], have been successfully illustrated by using zebrafish animal models.

#### 4.2.3. 2,3,7,8-Tetrachlorodibenzo-*p*-Dioxin (TCDD)

2,3,7,8-Tetrachlorodibenzo-*p*-dioxin (TCDD) is a halogenated aromatic hydrocarbon compound and is normally released into the environment via organic synthesis and burning of organic materials [184]. It is a potent developmental toxicant and endocrine disruptor [185]. Studies have shown its reproductive toxicity manifested by altered gonad development [186], reduced egg production and survival rate of eggs and fry [187], and decreased serum estradiol and vitellogenin level [188].

Several estradiol-biosynthesis genes such as* cyp19a1a*,* cyp11a1*, and* star* have been pointed out as the potential gene suppression targets of TCDD [189]. Meanwhile, downregulation of gonadotropin receptors and three estrogen receptors (*esr1*,* esr2a*, and* esr2b*) in the ovaries of adult zebrafish was observed [189]. On the other hand, aryl hydrocarbon receptor (AHR) signaling cascade appears as one of the major gene suppression pathways induced by TCDD [189]. The steroidogenesis disruption potential of TCDD is expressed by first binding to the AHR. The resulting AHR complex dimerizes with aromatic hydrocarbon receptor nuclear translocator (ARNT) protein in nucleus. Gene suppression in ovary is then observed following binding of the heterodimer complex to the aryl hydrocarbon-response element (AHRE) on genes, leading to disruption of estradiol biosynthesis in adult female ovaries [189]. Depressed gonadotropin responsiveness and estradiol biosynthesis have resulted in damaged ovaries with retarded follicular maturation and ovarian functions, which further results in reduction of egg released and spawning activities [190, 191].

Despite the absence of testicular lesion in TCDD-exposed male zebrafish, males seem to have contributed more to TCDD-induced reproductive toxicity, which are mainly manifested by reduced number of eggs spawned and amount of fertilized eggs [185, 192]. On top of that, it is important to note that offspring from fish exposed to TCDD experience reduced reproductive capacity too [185]. Conclusively, exposure to TCDD, especially during early life stages, brings adverse reproductive effects to both male and female zebrafish. It is important to note that the reproductive responses of zebrafish to TCDD are highly relevant to human following the high structural and functional similarities of the estrogen receptors in both zebrafish and humans and elucidation of perturbation of steroidogenesis regulation through AHR-dependent manner in TCDD-treated mammals [193–195].

#### 4.2.4. Di(2-Ethylhexyl) Phthalate (DEHP)

Di(2-Ethylhexyl) phthalate (DEHP) is a commonly used plasticizer. Although it can be readily degraded by microorganism, continual releases of large chemical volume into atmosphere following plastic manufacture, burning activities, and waste water effluents have led to substantial concentration in aquatic system [196, 197]. Recently, its abilities to bind to estrogen receptor and contributions to reproductive toxicity in aquatic life and mammals have been discovered. Several studies were carried out on zebrafish to further evaluate the reproductive effect of DEHP [159, 198, 199].

In adult male zebrafish which received intraperitoneal injection of DEHP, impaired spermatogenesis with accumulation of spermatogonia in testes were observed [198, 199]. Additionally, DEHP is capable of inducing oxidative stress in testes with consequent increase in spermatozoa DNA fragmentation [198, 199]. Sharp decline in embryo production was also observed following the blockage of male hormone synthesis by DEHP. With the absence of male pheromones in water, female egg depositions followed by sperm release are inhibited [199]. While for adult female zebrafish, impaired oocyte maturation and ovulation were the main toxicological effects of DEHP identified [159]. Dose-related effects were observed on both of the defects with maturation signals from membrane progestin receptors *β* (mPR*β*) and lhr greatly affected by low dose and ovulation signal from prostaglandin-endoperoxide synthase 2 (ptgs2) following high dose exposure [159]. Increased circulating level of bone morphogenetic protein-15 (BMP15), a hormone regulator which prevents precocious oocyte maturation, was suggested as one of the factors which contributes to disrupted oocyte maturation [159]. At the same time, suppressed expression of mPR*β* following increased level of BMP15 contributed to the lack of egg production in DEHP-exposed zebrafish [159, 200].

#### 4.2.5. Other Environmental Chemicals

In fact, zebrafish are increasingly used as powerful alternative model for assessing reproductive toxicity of a wide range of environmental chemicals as listed in Table 2. Most of the chemicals are industrial wastes and display high bioaccumulation factor. Disrupted gonad functions, altered steroidogenesis [75, 201, 202], and reduced quantities and qualities of germ cells along with low fertilization rate [201, 203, 204] are some of the reproductive toxicity effects observed in zebrafish chemical exposures (see Table 2).

As a whole, these findings provide strong rationales for conducting assessment on the reproductive toxicity of chemicals by using zebrafish. It was observed that most of the environmental chemicals disrupt the sexual functioning via perturbation of normal hormonal regulation of reproductive system. In view of the striking homologies in the endocrine regulation of reproduction as mentioned, there will be high relevance and predictability of chemical reproductive response between zebrafish and humans. The reproductive toxicity profile of the environmental chemicals established by using zebrafish animal model will be robust for uncovering the chemical-induced effects as well as appropriate protective approaches against chemical toxicity in human.

### 4.3. Environmental Induced Infertility

In the past few decades, effects of several environmental factors such as oxygen availability and exposure to exogenous heat on reproductive function have become of interest following the increase in the number of men who work in high altitude as well as in working areas with high heat exposure [205–207]. The reproductive effects of low oxygen availability in aquatic system, which are mainly due to eutrophication and organic pollution, have been extensively investigated in a range of fish, including zebrafish [28, 208–211]. Instead of causing direct cell damages in the reproductive organs, studies have discovered the negative indirect reproductive effect of hypoxia through alteration of circulating plasma sex steroid levels, notably testosterone and estradiol with underlying genetic and molecular mechanisms involving the expression of HPG-related genes [212], hypoxia-inducible factor 1 (HIF-1) [211, 212], cellular lipids and steroid hormones [210, 212], and leptin [211]. Hormonal imbalance eventually leads to a lag in gonadal growth, masculinization of the ovary, sex ratio distortion in favor of males, and arrest in gametogenesis [28, 211]. Prominent reduction or complete absence of ovulating females observed under hypoxic condition correlates with both of the changes in steroid and contractile gene expression. On top of that, fertility defects caused by hypoxia are further implicated by aberrant primordial germ cell (PGC) migration [213]. Collectively, despite the need for further elucidation of mechanisms underlying hypoxia-induced reproductive defect, the potential reproductive impairment of hypoxia in terms of abnormal gonadal development, reduced germ cell quantities and qualities, fertilization and hatching success, and larval and juvenile viability has been successfully revealed through the utilization of zebrafish as the animal model.

On the other hand, temperature of testicles is one of the critical factors which determines the sperms' quality and quantity in humans and mammals [214, 215]. Testicular temperature of approximately 2 to 4°C lower than body temperature is required for normal testicular function. Basically, the temperature is regulated via two mechanisms: the dissipation of heat through the surface of scrotum and the heat lost from incoming arterial blood to outgoing venous blood [216]. Through zebrafish study, anomalies in chromosomal number of the sperms were observed following the increase in water temperature [217]. The germ cell aneuploidy is mainly due to mutation of monopolar spindle 1 (Mps1), the critical mitotic checkpoint kinase factor [217]. As mentioned, sexual differentiation in some teleost can also be overridden by water temperature. Meanwhile, the influence of surrounding temperature on gonadal fate is also quite common among reptiles [218]. Masculinizing effect of high water temperature often related to the induced oocyte apoptosis and differentiation of spermatogonia as well as suppressed activity of gonadal aromatase [27, 219]. Moreover, hatching rhythm is temperature-sensitive, with shorter hatching rate observed at constant water temperature of 28°C as compared to lower temperature of 24°C and thermocycles [220].

## 5. Overall Limitations

Zebrafish has only emerged over the past decade as a research model in reproductive field [79, 221]. As a result, there is a definite lack of detailed information on its reproductive system as compared to other well-developed higher vertebrate model organisms. For instance, there is still significant gap in our understanding of the underlying mechanisms in stress-mediated infertility, especially on male reproductive system [150]. Additionally, more efforts are also needed to clarify the molecular genetic basis involved in zebrafish sex determination [222].

Additionally, zebrafish practice external insemination [1]. One of the major shortcomings of this reproduction mode is the dilution of gametes concentration required for successful fertilization [223, 224]. However, zebrafish display evolutionary development in their mechanism of sperm releases. Instead of staying close to the females and directly releasing sperms into the water column as described in other fishes that display external insemination [225], sperm trails are first laid by male zebrafish onto the substrates' surfaces [41]. Mucosubstances secreted by seminal vesicles are probably acting as the adhesive material in which the sperms are embedded in [41]. The production of sperm trails allows the release of active sperms over prolonged period of time, even after the males leave the spawning area, thus promoting egg insemination [41].

## 6. Conclusion

Despite the small size of zebrafish, the high similarities in reproductive functions and regulations between this small fish species and mammals have promoted them as the promising model in infertility research. Together with their biological advantages such as optical transparency during embryonic stage and rapid development, the utilization of zebrafish as model system has enabled us to delve more deeply and broadly into the reproductive functions. Additionally, our knowledge on the factors of infertility has been enriched by the researches of zebrafish. Human reproductive health risk assessment can thus be derived from the demonstration of underlying mechanisms associating infertility that are common between mammals and zebrafish. Most importantly, in-depth understanding about the underlying mechanisms leading to infertility contributes to the discovery and development of more effective fertility medications and technologies. Taken together, this review has highlighted the potential of zebrafish as valuable and reliable alternative model for studies aimed at answering questions concerning the reproductive functions as well as mechanisms of infertility in vertebrates.

## Figures and Tables

**Table 1:** The inhibitory effects of stress hormones (CRH, ACTH, and cortisol) on mammalian and zebrafish HPG axis.

Stress hormones	Inhibitory effects on HPG	References
*Mammal*		

CRH	(i) Inhibitory action on GnRH and testosterone release from rat hypothalamus and male *Rhesus macaque* (ii) CRH suppresses LH secretion in females and has dose-dependent inhibition effect on androgen production from human ovarian thecal cells as well	[226–228]

ACTH	(i) The dampening effect of ACTH on the responsiveness of the pituitary gland to GnRH and delayed estradiol-induced LH surge (ii) Direct actions of stress hormones on testosterone biosynthesis in Leydig cells	[229–231]

Cortisol	(i) Glucocorticoid inhibits the release of GnRH as well as LH and FSH via glucocorticoid-gonadotropin inhibitory hormone interaction in males (ii) Suppression of testosterone release from male rats under stress (iii) In females, cortisol acts on the HPG axis by inhibiting the release of GnRH, LH, and steroid hormones (estradiol and progesterone)	[232–234]

*Zebrafish*		

ACTH	(i) Reduced estradiol synthesis from zebrafish ovarian follicles (ii) Causes DNA damage, reduced amount of autophagosomes, and vacuolization of zebrafish follicles (iii) Elevated ACTH and cortisol following estradiol treatment are most likely contributed to low fertilization success	[150–152]

Cortisol	(i) Similar to ACTH, cortisol has adverse effects on female gametogenesis by causing DNA damage	[151]

**Table 2:** The reproductive effect of chemicals in zebrafish.

Chemicals	Chemical toxicity effect	References
Ammonium perchlorate	Reduced spawn volume	[235, 236]

Bisphenols	Skewed sex ratio in favor of females, imbalance of steroid hormones, reduced germ cell count, decreased hatching rates, and embryonic malformation	[203, 237]

Brominated flame retardants (e.g., 2,4,6-tribromophenol and 2,4-dibromophenol)	Skewed sex ratio, decreased fecundity, altered transcription of steroid genes and plasma concentration of sex hormones, disturbed gonad morphology, and complete hatching failure at high chemical dosage	[53, 238, 239]

Cobalt	Sperms with damaged DNA and reduced fertilization and embryo survival rates	[240]

2,4-Dichlorophenol	Altered steroid gene expression and plasma sex hormone level and reduced number of eggs released and hatching rate	[201]

Ethinyl estradiol	Reduced or complete failure of fertilization, reduced adult fecundity and vitellogenic response, abnormal vitellogenin induction, discernible effects on secondary sexual characteristics, altered sexual differentiation process, and degenerative sign of reproductive organs	[241–243]

Fluorotelomer alcohols	Reduced eggs and sperms production, affected steroidogenesis along with altered plasma reproductive hormones level, and reduced hatching rates	[202, 244]

Pharmaceutical drugs	Negative impacts on several reproductive parameters: courtship behaviour, number of egg spawned, hatching success, HPG gene transcription and hormone level, and gonad histological changes along with germ cells qualities	[245–248]

Polychlorinated biphenyls	Reduced number of eggs released and fertilized, altered ovary histology, and skewed sex ratio	[204, 249, 250]

Polycyclic musks	Antiestrogenic effect	[251]
